# The zero impact of the Vehicle Inspection Program on public health in São Paulo, SP

**DOI:** 10.11606/s1518-8787.2020054001856

**Published:** 2020-08-18

**Authors:** Orlei Ribeiro de Araujo, Milena Corrêa Araujo

**Affiliations:** I Universidade Federal de São Paulo Instituto de Oncologia Pediátrica Grupo de Apoio ao Adolescente e à Criança com Câncer São PauloSP Brasil Universidade Federal de São Paulo. Instituto de Oncologia Pediátrica. Grupo de Apoio ao Adolescente e à Criança com Câncer (GRAACC). São Paulo, SP, Brasil; II Centro Hospitalar do Sistema Penitenciário Fundação do ABC São PauloSP Brasil Centro Hospitalar do Sistema Penitenciário. Fundação do ABC. São Paulo, SP, Brasil

**Keywords:** Traffic-Related Pollution, adverse effects, Vehicle Emissions, Vehicle Inspections, Pneumopathy, prevention & control, Cardiovascular Diseases, prevention & control

## Abstract

**OBJECTIVE:**

To analyze the impact of two interventions (implementation and suspension of mandatory vehicle inspection) on morbidity and mortality due to conditions related to air pollution, from 2008 to 2017.

**METHODS:**

Interrupted time series (ARIMA models), using data available in public repositories.

**RESULTS:**

A total of 229,337 children of up to 5 years old were hospitalized due to respiratory diseases, and 1,053 died (average monthly mortality ratio for this population: 1.12/100,000). Exact 137,876 individuals over 40 years old were hospitalized for an acute myocardial infarction, and 19,492 died (3.7/100,000). A total of 11,010 individuals over 40 years old were hospitalized with malignant neoplasms of the respiratory system; 2,898 died (0.5/100,000). A total of 20,807 individuals over 60 years old were hospitalized with chronic obstructive pulmonary diseases; 2,627 died (1.5/100,000). As for strokes, 69,180 individuals were hospitalized, and 10,866 died (2.1/100,000). We found no significant regression coefficient for the implementation or suspension of the program regarding hospitalizations and deaths. 38,207 children of up to 14 years old were hospitalized with asthma, and 25 of them died (0.007/100,000). The coefficients show a monthly increase of 0.05 deaths/100,000 people (p = 0.01) in the post-inspection period. We found no correlation between the measured concentrations of the pollutants PM_2.5_ and CO – in a monitoring station, in the central region of the municipality – and the implementation or suspension of the inspection.

**CONCLUSIONS:**

No evidence confirms that the program had a measurable beneficial impact on morbidity and mortality due to respiratory and circulatory diseases.

## INTRODUCTION

In many urban areas, motor vehicles emissions have become a major source of air pollutants, such as carbon monoxide and dioxide, volatile organic compounds or hydrocarbons, nitrogen oxides, and particulate matter^1^. Fine particulate matter (PM_2.5_) – dust, smoke, and other matters with aerodynamic diameter less than or equal to 2.5 μm – are inhalable and can penetrate deep into the lungs. Ultrafine particles, with diameters less than 0.1 to 0.2 μm, have higher mass-concentration of adsorbed or condensed toxic pollutants per unit. Fine and ultrafine particulate matters are generated mainly from the burning of fossil fuels^2^. Wu et al.^3^ showed that long-term exposure to moderate PM_2.5_ levels (8 to 10 μg/m^3^) increases overall mortality by 2.8% in comparison to lower levels.

In Brazil, vehicle pollution control programs have led manufacturers to adopt technologies to reduce pollutant emissions. This effective medium-term measure tends to be compensated by the increase in vehicle fleet and their intensive use, as well as their ageing^4^. The metropolitan region of São Paulo, from 1996 to 2009, experienced a trend of annual decrease in all pollutants, except ozone, resulting from the intensive use of fossil fuels and biofuels (ethanol and biodiesel)^5^. Grounded on health concerns, in 2008, the municipality of São Paulo passed a legislation making motor vehicle inspection mandatory for controlling pollutants emission – the Vehicle inspection and maintenance (I/M-SP) program^6^. The program (funded largely from public monies) was mandatory for all vehicles as of January 2010, and 15,481,355 vehicles were inspected until its suspension, on January 31, 2014^7^. Its implementation and suspension provide an opportunity for a temporal analysis of its potential impacts on public health. To meet Bradford-Hill’s criteria of temporality and causal inference, the improvement in morbidity indicators temporally correlated with the implementation of the inspection should disappear after its interruption^8^. This study aimed to analyze potential impacts of the two interventions – the implementation of mandatory inspection and its subsequent suspension – on morbidity and mortality due to conditions commonly associated with air pollution.

## METHODS

This is an interrupted time series study that used data available in public repositories. Common diseases caused by air pollution were searched in DATASUS/Tabnet database^9^, such as: asthma in children under 14 years old (International Classification of Diseases [ICD] J45); respiratory diseases in children under 5 years old (ICD J00–J99); pneumonia in patients older than 60 years (ICD J18); bronchitis, emphysema, and chronic obstructive pulmonary disease in patients older than 60 years (ICD J40–J44); ischemic heart diseases (ICD I20–I25) and vascular accidents (ICD I64) in adults older than 40 years; and malignant neoplasms of the trachea, bronchi, and lungs (ICD C33–C34) in individuals older than 40 years. Data on hospital morbidity and mortality (number of hospitalizations and deaths by disease and age group), for all months from January 2008 to December 2017, were collected from the Hospital Information System of the Brazilian Unified Health System (HIS/SUS). For demographic data, we used the numbers referring to the population living in the municipality, disclosed by the Interagency Network of Health Information– a cooperation between the Ministry of Health, the Pan American Health Organization, the Brazilian Institute of Geography and Statistics (IBGE), and other institutions in an effort to standardize population estimates by municipality, age, and gender, in the period ranging from 2000 to 2015^10^. Considering the lack of estimates for 2016 and 2017, Prais-Winsten method was used to quantitatively estimate growth rate for different age groups, by calculating the annual percent change (APC), based on the previous ten years^11^.

**Figure 1:**
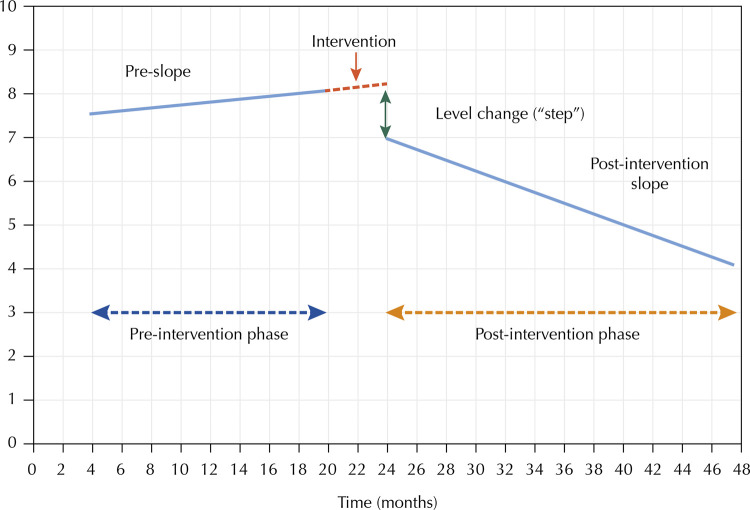
Standardized effects of an intervention (level and slope change) on a hypothetical model of prognosis variation (Y axis) in a given period (X axis).

To construct the time series, the years of 2008 and 2009 were considered as the 24 months pre-intervention. January 2010 was considered as the first month of the first intervention (large-scale inspection for all vehicles), and January 2014 as the first month of the second intervention (inspection suspension); the analysis covered all months until December 2017. We adopted the interrupted time series method for statistical analysis, which consists in data collection at multiple points in time, before and after an intervention, and pre- and post-trend verification. ARIMA (autoregressive integrated moving average)^12^models were constructed, considering trends and autocorrelations for estimating interventions effect. This analysis obtains estimates for the regression coefficients related to the magnitudes of two standardized effects: a level change (also called “step” or “step change”), and a trend change before and after intervention. Level change is the difference between the level observed at the first moment of intervention and the level predicted by the temporal trend before the intervention; trend change is the difference between the pre- and post-intervention slopes^13^. A significant negative change in the post-intervention level and slope would indicate a reduction in the assessed rates (Figure 1)^14^.

For the ARIMA analysis, prognoses (relative frequencies of hospitalizations and deaths: number × 100,000/population of the age group in the year) were defined as dependent variables, whereas “time” (months), “phase” (0: pre-intervention and 1: post-intervention), and “interval” (all pre-intervention months were deemed as 0, and post-intervention months follow normal numbering) were defined as independent variables. The β coefficient for “time” (β1) provides the slope of the pre-intervention regression line; for “phase” (β2), the coefficient provides the change in the intercept; as for “interval” (β3), the coefficient provides the difference between the pre- and post-intervention slope^14^. The regression model is:

Prognose = constant + β1.time + β2.phase + β3.interval

Variables named “pre” and “post” were created for the time points of interest – 6, 12, 24, 36, and 48 months, – moving the slope lines to the given points. For example, in an ARIMA analysis targeting “phase” and applying “pre-6” and “post-6” as independent variables, and “deaths” prognosis as dependent variable, the coefficient for “phase” would provide the intervention effect on the mortality rate after six months. Statistical analyses were performed using SPSS 20.0.

## RESULTS

During the 120-month period from January 2008 to December 2017, DATASUS recorded 229,337 hospitalizations for respiratory diseases in children up to 5 years old, with an average length of stay of 5.9 days. In total, 1,053 deaths were reported (average monthly mortality ratio for this population: 1.12/100,000). Table 1 shows the coefficients of ARIMA models for these diseases. We found no statistically significant coefficient, neither for the implementation of the inspection nor for its suspension, related to the number of hospitalizations and deaths.

**Table 1:** ARIMA model for hospitalizations and deaths from respiratory diseases among children up to 5 years old in the 10-year period.

Hospitalizations for respiratory diseases among children under 5 years old							Deaths from respiratory diseases among children under 5 years old					
Inspection implementation				Inspection suspension			Inspection implementation			Inspection suspension		
	β	95%CI (β)	p	β	95%CI (β)	p	β	95%CI (β)	p	β	95%CI (β)	p
Time	1.02	-4.7–6.9	0.79	0.32	-7.8–8.4	0.95	0.00	-0.02–0.02	0.77	0.00	-0.02–0.03	0.80
Interval	-2.06	-9.4–5.3	0.64	-0.99	-10.5–8.5	0.86	-0.01	-0.02–0.02	0.61	-0.01	-0.04–0.02	0.65
Phase (6 m)	21.20	-92.1–134.5	0.76	8.31	-127.6–144.2	0.92	-0.16	-0.6–0.27	0.54	0.01	-0.52–0.54	0.98
Phase (12 m)	22.90	-96.4–142.2	0.75	-11.03	-151.2–129.1	0.90	-0.18	-0.7–0.3	0.54	-0.10	-0.7–0.51	0.79
Phase (24 m)	28.83	-93.6–151.2	0.70	-23.41	-163.7–116.9	0.78	-0.18	-0.8–0.4	0.61	-0.24	-0.94–0.45	0.56
Phase (36 m)	33.27	-88.3–154.9	0.65	-24.02	-162.9–114.8	0.77	-0.15	-0.8–0.4	0.67	-0.30	-1.02–0.42	0.49
Phase (48 m)	35.88	-84.6–156.3	0.62	-23.28	-161–114.4	0.78	-0.13	-0.7–0.5	0.72	-0.31	-1.04–0.42	0.48

β: regression model coefficient; 95%CI (β): 95% confidence interval of the coefficient; p: significance; m: months

Between January 2008 and December 2017, 38,207 hospitalizations for asthma were reported among children of up to 14 years old; 25 resulted in death (average monthly mortality ratio for this population: 0.007/100,000); the average length of hospital stay was 3.5 days. Table 2 shows ARIMA models coefficients for this pathology. We found no statistically significant coefficient related to the number of hospitalizations, neither for the implementation of the inspection nor for its suspension. As for deaths, data suggest an increase from 0.03 (at 24 months) to 0.05 deaths/100,000 people (at 48 months) after inspection was implemented. We found no significant coefficient after its suspension.

**Table 2:** ARIMA model for hospitalizations and deaths from respiratory diseases among children up to 14 years old in the 10-year period.

Hospitalizations for asthma among children under 14 years old							Deaths from asthma among children under 14 years old					
Inspection implementation				Inspection suspension			Inspection implementation			Inspection suspension		
	β	95%CI (β)	p	β	95%CI (β)	p	β	95%CI (β)	p	β	95%CI (β)	p
Time	-0.17	-0.42–0.07	0.28	0.02	-0.12–0.17	0.78	0.00	-	0.93	0.00	-	0.07
Interval	0.11	-0.17–0.39	0.50	-0.19	-0.35 – -0.03	0.06	0.00	-	0.67	0.00	-	0.05
Phase (6 m)	2.57	-2.46–7.6	0.40	2.87	-0.04–5.77	0.10	0.01	-0.02–0.03	0.66	0.01	-0.01–0.01	0.36
Phase (12 m)	3.23	-2.34–8.8	0.34	1.63	-1.63–4.88	0.41	0.02	-0.01–0.04	0.30	0.01	-0.01–0.02	0.32
Phase (24 m)	3.81	-2.3–9.9	0.30	0.94	-2.58–4.46	0.66	0.03	0.01–0.06	0.04	0.01	-0.01–0.02	0.48
Phase (36 m)	3.83	-2.4–10.1	0.31	0.98	-2.63–4.59	0.65	0.04	0.01–0.07	0.01	0.00	-0.01–0.02	0.68
Phase (48 m)	3.73	-2.5–9.96	0.32	1.19	-2.43–4.8	0.59	0.05	0.02–0.07	0.01	0.00	-0.01–0.01	0.83

β: regression model coefficient; 95%CI (β): 95% confidence interval of the coefficient; p: significance; m: months

For malignant neoplasms of the respiratory and intrathoracic organs in individuals older than 40 years, 11,010 hospitalizations were analyzed, among which 2,898 resulted in death (average monthly mortality ratio for this population: 0.54/100,000); the average length of hospital stay was 8.2 days. The ARIMA models showed no significant coefficient for hospitalization numbers related to the inspection implementation or suspension. As for deaths, the coefficients suggest a reduction of 0.14 deaths/100,000 in the first six months after implementation. It reflects a pre-inspection trend, as the “time” coefficient is also significant, with a reduction of 0.01 death/100,000 per month. After this period, or after the suspension of the inspection, no significant coefficient was observed.

A total of 137,876 hospitalizations due to acute myocardial infarction (AMI) among individuals older than 40 years were reported; 19,492 resulted in death (average monthly mortality ratio for this population: 3.7/100,000); the average length of hospital stay was 9.2 days. We found no correlation with the implementation or suspension of the inspection, nor statistically significant variation in the time series.

Exact 69,180 hospitalizations due to stroke were analyzed, with an average length of stay of 9.2 days. Among this number, 10,866 deaths were reported (monthly ratio 2.1/100,000 for this population). We observed no significant regression coefficient related to the implementation or suspension of the inspection.

As for chronic obstructive pulmonary diseases in patients aged 60 years or older, 20,807 hospitalizations were recorded in 10 years, with an average length of stay of 9.6 days. 2,627 of these cases resulted in death, with a monthly ratio coefficient of 1.5/100,000 for this population. We found no correlation between the rates and the implementation or suspension of vehicular inspection.

**Figure 2:**
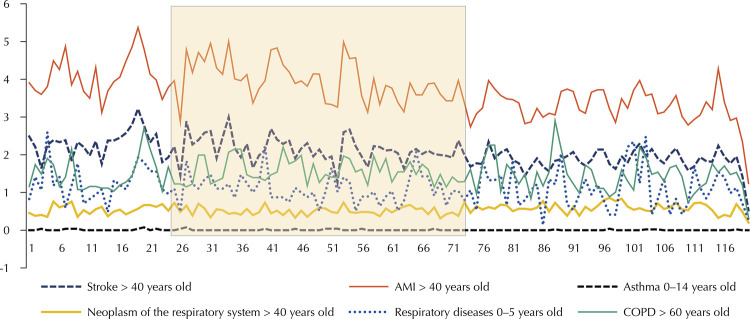
Temporal evolution of monthly mortality ratio for the age group, by pathologies. The inspection program period is highlighted.

Figure 2 shows the temporal evolution of mortality coefficients.

**Figure 3:**
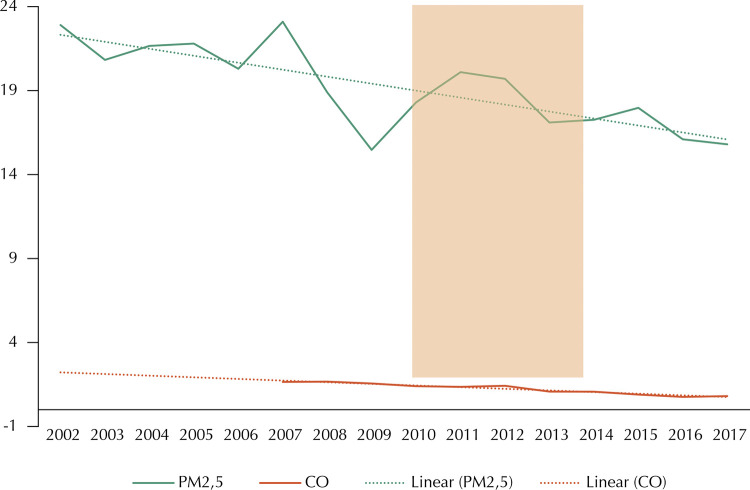
Temporal trend of PM2,5 (in μg/m3 ) and carbon monoxide (CO, in ppm, after 2007) concentrations according to Cerqueira César monitoring station, in the central region of the city of São Paulo. Observation: Trend lines (dashed) show the downward slope trend before the inspection period (shaded).

The main aim of this study was to analyze morbidity and mortality. We also created an ARIMA model for PM_2.5_ and carbon monoxide (CO) pollutants. Such a model presents data from the Cerqueira César monitoring station, in the central region of the municipality of São Paulo – the only with continuous data from 2003 to 2017 in the report of the Environmental Company of São Paulo State (CETESB)^4^, enabling the time series analysis. For PM_2,5_, we observed a -0.65 “time” coefficient (standard error = 0.22; p = 0.02) by the time the inspection was implemented, showing that PM_2.5_ concentration was decreasing 0.65 μg/m^3^ yearly before inspection. The Cerqueira César station showed no significant correlation between the trends of PM_2.5_ and carbon monoxide concentrations and the implementation or suspension of the inspection (Figure 3).

## DISCUSSION

Our results showed that the Vehicle inspection and maintenance (I/M-SP) program in the city of São Paulo had no measurable impact on hospital morbidity and mortality due to respiratory and circulatory diseases among the studied age groups. A monitoring station installed in the central area of the city also revealed no effect on atmospheric levels of PM_2.5_ and carbon monoxide.

The disorderly urbanization in São Paulo and the increasing sources of air pollution have led the city to a critical situation, with pollutants concentrations often exceeding the values required for minimum quality^15^many efforts have been made to describe the emissions sources and to analyse the primary and secondary formation of pollutants under a process of increasing urbanisation in the metropolitan area. From the occurrence of frequent violations of air quality standards in the 1970s and 1980s (due to the uncontrolled air pollution sources. The Vehicle Inspection and Maintenance program was shrouded in controversy, being accused of hiring the company responsible for implementation without the bid and even transferring large public funds, as well as lacking transparency in its practices and results. According to the last published report, in 2012, the company projected a 0.96 μg/m^3^ reduction in PM_2.5_ concentration, enabled by diesel vehicles inspection, which would avoid 559 deaths due to respiratory diseases^16^. Such projections, originated in a report from the Universidade de São Paulo, were used as propaganda and spread in the press: Saldiva et al.^17^ estimated that by 2011, with 75% of the fleet inspected and adequate, there would be a 28% reduction in PM_2.5_ issued by diesel vehicles, resulting in less 1.18 μg/m^3^ in the concentration levels of the municipality. To estimate the impact of the on mortality, the researchers used another projection mapped by the World Health Organization – which considers a 6% impact on mortality for each 10 μg/m^3^ increase in particulate matter concentration – reaching 379 spared deaths and 894 spared hospitalizations in the municipality, only by inspecting 75% of the diesel fleet^17^. These results are only estimates, without measurements or observations groundings. In 2011, the city of São Paulo reported 5,052 deaths from respiratory causes, at all age groups^9^. The 379 spared deaths impact (7.5%) would not go unnoticed in case it occurred.

If PM_2.5_ levels remain the same as in 2011, 250,000 pollution-related deaths are expected in the state of São Paulo by 2030. The most affected context is believed to be circulatory and respiratory diseases among older adults – the fastest-growing and more vulnerable demographic group, as well as those under 5 years old^18^. The time series analyzed showed no significant variation in respiratory diseases among children under 5 years during the 10-year period. Regarding hospital mortality due to asthma among children under 14 years old, we observed an upward trend from 2011 to 2014. The increase in the number of deaths attributable to asthma may be related to pollutants, but, more often, it reflects the lack of access to appropriate medications and therapies^19^. As we observed no change after inspection suspension, it is more likely a temporal coincidence.

We opt to analyze subpopulations by age groups, considering the already established risks in numerous studies. Children and older adults are populations particularly susceptible to air pollutants exposure with greater evidence of complications^20^. To select the analyzed pathologies, we chose those related to respiratory diseases – such as asthma and chronic obstructive pulmonary diseases – and particulate matter. The pathophysiological relationship between exposure to particulate matter and cardiovascular diseases as infarction and strokes entails oxidative stress, with markers such as oxidized lipids, in addition to inducing a prothrombotic state: exposure induces the production of fibrinogen, von Willebrand factor, and other coagulation factors, and activates antifibrinolytic pathways, increasing plasma viscosity and activating platelets. Besides being associated with endothelial dysfunction^21^, short- and long-term PM_2,5_ exposure also entails strong epidemiological correlations. Gold et al.^22^ showed a 0.5% to 1.5% increase in the risk of cardiovascular diseases for every increase of 5 to 6 μg/m3 in PM_2.5_ concentrations. Acute exposure to PM_2.5_ may increase the risk of death from cardiovascular diseases by 69%^22^. Exposure to high levels of carbon monoxide and PM_2,5_ may also raise blood^20^ pressure, potentiating the risk of strokes. A meta-analysis showed an increased relative risk of death from lung cancer with chronic exposure to PM_2.5_, although no relationship with incidence was found^2^.

Inspection and maintenance programs focused on “reducing vehicle emissions” tend to fail systematically in cities in which car is the predominant mode of individual transport, due to the difficulty in controlling its three aspects: vehicle, fuel, and traffic. Guangzhou, a Chinese city with similar urban density to São Paulo, reported no improvement in air quality, particularly regarding PM_2.5_ and ozone, in two decades of these programs. Similarly to São Paulo, public policies stimulated and facilitated the purchase of individual vehicles, resulting in average PM_2.5_ concentrations of 49 μg/m3 – four times higher than recommended^24^. In Brazil, the federal initiative *Programa de Controle da Poluição do Ar por Veículos Automotores* (PROCONVE – Air Pollution Control Program for Vehicles) was the main responsible for reducing air pollutants emission. PROCONVE was initiated in phases up to 1988, adopting the US Environmental Protection Agency (USEPA) parameters. It achieved results by establishing emission limits for the industries regarding national vehicles, as well as encouraging the use of biofuels (such as ethanol and biodiesel) and the reduction in fuel sulfur.^15^ In São Paulo, the decrease in PM_2.5_ and carbon monoxide concentrations up from 2000 can be attributed both to these initiatives and to the fleet renewal;^4^Figure 3 shows the trends.

The plainest reason why I/M-SP had no impact on particulate matter levels in the central region and on morbidity and mortality rates is stressed in the responsible company report: of the 3,037,474 vehicles inspected in 2012 – including motorcycles, cars, buses, diesel light vehicles, and trucks, – 97.3% were approved at first inspection. That is, for every 100 vehicles, only three would indeed need to be inspected by the adopted criteria, elucidating the irrelevance of the service^16^. It is unknown whether failed or uninspected vehicles were removed from circulation, which could be an effective measure, although limited – radically removing inappropriate vehicles and motorcycles would have but a transient effectiveness, compensated in few years by the fleet growth^25^.

After costing hundreds of millions of Brazilian reais to the municipality population, the I/M-SP had a melancholic end. The São Paulo Public Prosecutor’s Office filed a claim against the municipal managers and the supplier for “technical, economic, and financial incapacity to execute the contract; fraud in changing shareholding control and composing the share capital; unconstitutional municipal legislations on mandatory vehicle inspection; and several others irregularities”^26^. Even if the inspection in São Paulo could tailor all cars and cease the circulation of vehicles outside the specifications, the massive burning of fossil fuels and the traffic jam would still pose problems. According to CETESB, the current situation of pollution in São Paulo requires complementary measures that enable reducing motorized trips and traffic jam, improving non-polluting public transport supply, and increasing the efficiency of public transport by buses and freight transport, as well as improving road network management with the support of land-use planning to reduce the impact of mobility and logistics^4^. All resources applied to the unnecessary vehicle inspection could be allocated to public health measures, by distributing inhaled corticosteroids and ensuring regular follow-up for asthmatics, or assistance programs for risk groups of specific morbidities.

This study is subject to some limitations regarding its retrospective and observational nature. Although statistical models might accurately represent reality, this type of study hinders the control over patients’ characteristics and computing confounding variables. Data stored from multiple sources in public repositories might implicate heterogeneous quality. However, the enormous amount of data available in DATASUS enabled us to create a model that securely reflects the non-stationary temporal behavior of the analyzed series, providing a potential usefulness for its application in evaluating new public policies. To evaluate the performance of any health policy, we must consider the efficiency, transparency, and publicity of the data.
