# Supplementary material for: The zero impact of the Vehicle Inspection Program on public health in São Paulo, SP
Source: Rev Saude Publica. 2020 Aug 18;54:84. doi: 10.11606/s1518-8787.2020054001856 (PMC7438032; doi:10.11606/s1518-8787.2020054001856)
Supplement: Translation

# Translation: O impacto nulo do Programa de Inspeção Veicular na saúde pública em São Paulo, SP

**Authors:** Orlei Ribeiro de Araujo, Milena Corrêa Araujo

**Affiliations:** I Brasil Universidade Federal de São Paulo. Instituto de Oncologia Pediátrica. Grupo de Apoio ao Adolescente e à Criança com Câncer (GRAACC). São Paulo, SP, Brasil; II Brasil Centro Hospitalar do Sistema Penitenciário. Fundação do ABC. São Paulo, SP, Brasil

**Keywords:** Traffic-Related Pollution, efeitos adversos, Emissões de Veículos, Verificação de Veículos, Pneumopatias, prevenção & controle, Doenças Cardiovasculares, prevenção & controle

## Abstract

**OBJETIVO:**

Analisar o impacto de duas intervenções (introdução e suspensão da inspeção veicular obrigatória) sobre a morbidade e mortalidade por condições associadas à poluição do ar no período de 2008 a 2017.

**MÉTODOS:**

Séries temporais interrompidas (método Arima), com dados disponíveis em repositórios públicos.

**RESULTADOS:**

Houve 229.337 internações por doenças respiratórias em crianças até 5 anos de idade, com 1.053 óbitos (coeficiente médio de mortalidade mensal para a população da idade: 1,12/100.000). Para infarto agudo do miocárdio em maiores de 40 anos, houve 137.876 internações, com 19.492 óbitos (3,7/100.000). Para as neoplasias malignas do aparelho respiratório em maiores de 40 anos, foram 11.010 internações, com 2.898 óbitos (0,5/100.000). Para doenças pulmonares obstrutivas crônicas em maiores de 60 anos, foram 20.807 internações, com 2.627 óbitos (1,5/100.000). Para os acidentes vasculares cerebrais, foram 69.180 internações e 10.866 óbitos (2,1/100.000). Não houve nenhum coeficiente de regressão significativo para a introdução ou suspensão do programa em relação às internações e óbitos. Para asma em crianças até 14 anos, houve 38.207 internações e 25 óbitos (0,007/100.000), e os coeficientes mostram aumento de 0,05 óbitos/100.000 pessoas por mês (p = 0,01) no período pós-introdução da inspeção. Em modelo para os poluentes MP_2,5_ e CO, em estação de monitoramento da região central do município, não houve correlação entre as concentrações medidas e a introdução ou a suspensão da inspeção.

**CONCLUSÕES:**

Não há evidências de que o programa tenha tido qualquer efeito benéfico mensurável sobre a morbidade e a mortalidade por doenças respiratórias e do aparelho circulatório.

## INTRODUÇÃO

Em muitas áreas urbanas, as emissões de veículos automotores se tornaram a principal fonte de poluentes do ar, incluindo monóxido e dióxido de carbono, compostos orgânicos voláteis ou hidrocarbonetos, óxidos de nitrogênio e materiais particulados^1^. Materiais particulados finos (PM_2,5_), constituídos por poeiras, fumaças e outros materiais com diâmetro aerodinâmico menor ou igual a 2,5 μm, são inaláveis e podem penetrar profundamente nos pulmões. Partículas ultrafinas, com diâmetros menores que 0,1 a 0,2 μm, têm maiores concentrações de poluentes tóxicos adsorvidos ou condensados por unidade de massa. Os materiais particulados finos e ultrafinos têm origem principalmente na queima de combustíveis fósseis^2^. No estudo de Wu et al.^3^, foi demonstrado que a exposição em longo prazo a níveis moderados de PM_2,5_ (8 a 10 μg/m^3^) causa um aumento na mortalidade geral de 2,8%, em relação à exposição a níveis menores que 8 μg/m^3^.

No Brasil, programas federais de controle da poluição por veículos têm levado os fabricantes a adotar tecnologias para redução da emissão de poluentes. É uma medida eficaz que tende a ser compensada em médio prazo pelo aumento da frota e uso intensivo dos veículos, além do seu envelhecimento^4^. Na região metropolitana de São Paulo, no período de 1996 a 2009, observou-se uma tendência de redução anual de todos os poluentes, com exceção do ozônio, devido ao uso intensivo de combustíveis fósseis e biocombustíveis (etanol e biodiesel)^5^. Baseando-se em preocupações sanitárias, o município de São Paulo instituiu em 2008 uma lei tornando obrigatória a inspeção de veículos automotores para controle da emissão de poluentes, o Programa de Inspeção e Manutenção de Veículos em Uso (I/M-SP)^6^. O programa, financiado em grande parte com recursos públicos, foi obrigatório para todos os veículos a partir de janeiro de 2010, sendo inspecionados 15.481.355 veículos até a interrupção em 31 de janeiro de 2014^7^. A introdução e a interrupção fornecem uma oportunidade de análise temporal dos possíveis efeitos sobre a saúde pública. Uma melhora nos indicadores de morbidade, correlacionada temporalmente à introdução da inspeção, deveria desaparecer após a interrupção, para satisfazer os critérios de temporalidade e inferência causal de Hill^8^. O objetivo deste estudo foi analisar possíveis impactos das duas intervenções (a introdução da inspeção obrigatória e sua posterior suspensão) sobre a morbidade e mortalidade por condições comumente associadas à poluição do ar.

## MÉTODOS

Trata-se de um estudo de séries temporais interrompidas com dados disponíveis em repositórios públicos. Foram pesquisadas no banco de dados Datasus/Tabnet^9^ as doenças comumente associadas à poluição do ar, como asma em menores de 14 anos (Classificação Internacional de Doenças [CID] J45); doenças respiratórias em menores de 5 anos (CID J00–J99); pneumonias em maiores de 60 anos (CID J18); bronquite, enfisema e doença pulmonar obstrutiva crônica em maiores de 60 anos (CID J40–J44); doenças isquêmicas do coração (CID I20–I25) e acidentes vasculares (CID I64) em maiores de 40 anos; e neoplasias malignas de traqueia, brônquios e pulmões (CID C33–C34) em maiores de 40 anos. Foram coletados do Sistema de Informações Hospitalares do Sistema Único de Saúde (SIH/SUS) os dados referentes à morbidade e mortalidade hospitalar (número de internações e número de óbitos por doença e faixa etária) no munícipio de São Paulo, em todos os meses de janeiro de 2008 a dezembro de 2017. Para os dados demográficos, utilizamos os números referentes à população residente no município disponibilizados pela Rede Interagencial de Informações para a Saúde (Ripsa), uma cooperação entre o Ministério da Saúde, a Organização Pan-Americana de Saúde, o Instituto Brasileiro de Geografia e Estatística (IBGE) e outras instituições, em um esforço para padronizar as estimativas populacionais por município, idade e sexo, no período de 2000 a 2015^10^. Como não havia estimativas disponíveis para os anos de 2016 e 2017, utilizamos o método de Prais-Winsten para estimar quantitativamente a tendência de crescimento para as faixas de idade, com o cálculo da *annual percent change* (APC), baseando-se nos dez anos anteriores^11^.

Para a construção das séries temporais, foram considerados os anos de 2008 e 2009 como os 24 meses pré-intervenção. O mês de janeiro de 2010 foi considerado como o primeiro mês da primeira intervenção (inspeção em larga escala, para todos os veículos), e janeiro de 2014 o primeiro mês da segunda intervenção (a suspensão da inspeção), levando-se a análise até dezembro de 2017. Para a análise estatística, utilizamos o método de séries temporais interrompidas, cuja característica são os dados coletados em múltiplos pontos antes e depois de uma intervenção, sendo verificadas as tendências pré e pós. Foram construídos modelos Arima (*autoregressive integrated moving average*)^12^, levando em consideração as tendências e autocorrelações para a estimativa do efeito das intervenções. Nessa análise, estimativas para os coeficientes de regressão correspondentes às magnitudes de dois efeitos padronizados são obtidas: uma mudança de nível (também chamada de “degrau” ou “*step change*”) e uma mudança na tendência antes e depois da intervenção. Uma mudança de nível é definida como a diferença entre o nível observado no primeiro momento de intervenção e o nível previsto pela tendência temporal pré-intervenção, e uma mudança na tendência é definida como a diferença entre as inclinações pós e pré-intervenção^13^. Uma mudança negativa significativa no nível e na inclinação pós indicaria uma redução nas taxas avaliadas (Figura 1)^14^.

**Figura 1:** Efeitos padronizados de uma intervenção (alteração de nível e de inclinação), em um modelo hipotético de variação de um prognóstico (eixo Y) em um determinado período de tempo (eixo X).

Na análise Arima, os prognósticos (frequências relativas de internações e óbitos: número × 100.000/população da faixa etária no ano) foram definidos como variáveis dependentes, e como independentes as variáveis “tempo” (meses), “fase” (0: pré-intervenção; 1: pós-intervenção) e “intervalo” (atribuindo-se o valor 0 para todos os meses pré-intervenção, enquanto os meses pós-intervenção seguem a numeração normal). Dessa forma, o coeficiente β para “tempo” (β1) fornece a inclinação da linha de regressão pré-intervenção, o coeficiente para “fase” (β2) fornece a alteração no intercepto, e o coeficiente para “intervalo” (β3) fornece a diferença na inclinação pré e pós-intervenção^14^. O modelo de regressão é:

Prognóstico = constante + β1.tempo + β2.fase + β3.intervalo

Variáveis denominadas “pré” e “pós” foram criadas para os pontos temporais de interesse (6, 12, 24, 36 e 48 meses), movendo as linhas de inclinação até os pontos determinados. Fazendo, como exemplo, uma análise Arima com “fase”, “pré-6” e “pós-6” como variáveis independentes e o prognóstico “óbitos” como dependente, o coeficiente para “fase” fornece o efeito da intervenção em seis meses sobre a taxa de óbitos. As análises estatísticas foram realizadas utilizando-se o software SPSS 20.0.

## RESULTADOS

No período de 120 meses de janeiro de 2008 a dezembro de 2017, o Datasus registrou 229.337 internações por doenças respiratórias em crianças até 5 anos de idade, com tempo médio de 5,9 dias. Foram registrados 1.053 óbitos (coeficiente médio de mortalidade mensal para a população da idade: 1,12/100.000). Os coeficientes dos modelos Arima para essas doenças estão na Tabela 1. Não houve nenhum coeficiente estatisticamente significante, tanto para a introdução quanto para a suspensão da inspeção, em relação ao número de internações e óbitos.

**Tabela 1:** Modelo Arima para internações e óbitos por doenças respiratórias em crianças até 5 anos de idade no período de 10 anos.

Internações por doenças respiratórias em menores de 5 anos							Óbitos por doenças respiratórias em menores de 5 anos					
Introdução da inspeção				Suspensão da inspeção			Introdução da inspeção			Suspensão da inspeção		
	β	IC95% (β)	p	β	IC95% (β)	p	β	IC95% (β)	p	β	IC95% (β)	p
Tempo	1,02	-4,7–6,9	0,79	0,32	-7,8–8,4	0,95	0,00	-0,02–0,02	0,77	0,00	-0,02–0,03	0,80
Intervalo	-2,06	-9,4–5,3	0,64	-0,99	-10,5–8,5	0,86	-0,01	-0,02–0,02	0,61	-0,01	-0,04–0,02	0,65
Fase (6 m)	21,20	-92,1–134,5	0,76	8,31	-127,6–144,2	0,92	-0,16	-0,6–0,27	0,54	0,01	-0,52–0,54	0,98
Fase (12 m)	22,90	-96,4–142,2	0,75	-11,03	-151,2–129,1	0,90	-0,18	-0,7–0,3	0,54	-0,10	-0,7–0,51	0,79
Fase (24 m)	28,83	-93,6–151,2	0,70	-23,41	-163,7–116,9	0,78	-0,18	-0,8–0,4	0,61	-0,24	-0,94–0,45	0,56
Fase (36 m)	33,27	-88,3–154,9	0,65	-24,02	-162,9–114,8	0,77	-0,15	-0,8–0,4	0,67	-0,30	-1,02–0,42	0,49
Fase (48 m)	35,88	-84,6–156,3	0,62	-23,28	-161–114,4	0,78	-0,13	-0,7–0,5	0,72	-0,31	-1,04–0,42	0,48

β: coeficiente do modelo de regressão; IC95% (β): intervalo de confiança de 95% do coeficiente; p: significância; m: meses.

Foram analisadas 38.207 internações por asma em crianças até 14 anos de idade, entre janeiro de 2008 e dezembro de 2017. Houve 25 óbitos (coeficiente médio de mortalidade mensal para a população da idade: 0,007/100.000), e o tempo médio de permanência hospitalar foi de 3,5 dias. Os coeficientes dos modelos Arima para essa patologia estão na Tabela 2. Não houve nenhum coeficiente estatisticamente significante em relação ao número de internações, tanto para a introdução quanto para a suspensão da inspeção. Quanto aos óbitos, os dados sugerem um aumento de 0,03 (com 24 meses) a 0,05 óbitos/100.000 pessoas (48 meses) no período pós-introdução da inspeção. Não se observa nenhum coeficiente significativo após a suspensão da inspeção.

**Tabela 2:** Modelo Arima para internações e óbitos por asma em crianças até 14 anos de idade no período de 10 anos.

Internações por asma em menores de 14 anos							Óbitos por asma em menores de 14 anos					
Introdução da inspeção				Suspensão da inspeção			Introdução da inspeção			Suspensão da inspeção		
	β	IC95% (β)	p	β	IC95% (β)	p	β	IC95% (β)	p	β	IC95% (β)	p
Tempo	-0,17	-0,42–0,07	0,28	0,02	-0,12–0,17	0,78	0,00	-	0,93	0,00	-	0,07
Intervalo	0,11	-0,17–0,39	0,50	-0,19	-0,35 – -0,03	0,06	0,00	-	0,67	0,00	-	0,05
Fase (6 m)	2,57	-2,46–7,6	0,40	2,87	-0,04–5,77	0,10	0,01	-0,02–0,03	0,66	0,01	-0,01–0,01	0,36
Fase (12 m)	3,23	-2,34–8,8	0,34	1,63	-1,63–4,88	0,41	0,02	-0,01–0,04	0,30	0,01	-0,01–0,02	0,32
Fase (24 m)	3,81	-2,3–9,9	0,30	0,94	-2,58–4,46	0,66	0,03	0,01–0,06	0,04	0,01	-0,01–0,02	0,48
fase (36 m)	3,83	-2,4–10,1	0,31	0,98	-2,63–4,59	0,65	0,04	0,01–0,07	0,01	0,00	-0,01–0,02	0,68
Fase (48 m)	3,73	-2,5–9,96	0,32	1,19	-2,43–4,8	0,59	0,05	0,02–0,07	0,01	0,00	-0,01–0,01	0,83

β: coeficiente do modelo de regressão; IC95% (β): intervalo de confiança de 95% do coeficiente; p: significância; m: meses.

Para as neoplasias malignas do aparelho respiratório e órgãos intratorácicos em maiores de 40 anos, foram analisadas 11.010 internações, com 2.898 óbitos (coeficiente médio de mortalidade mensal para a população da idade: 0,54/100.000), e o tempo médio de permanência hospitalar foi de 8,2 dias. Não há nenhum coeficiente significativo nos modelos Arima para os números de internação, em relação à introdução ou suspensão da inspeção. Quanto aos óbitos, os coeficientes sugerem uma redução de 0,14 óbitos/100.000 nos primeiros seis meses após a introdução da inspeção, refletindo uma tendência prévia à inspeção, pois o coeficiente para tempo também é significativo, com redução de 0,01 óbito/100.000 pessoas por mês previamente à introdução. Não se observa nenhum coeficiente significativo após esse período ou após a suspensão.

Para infarto agudo do miocárdio (IAM) em maiores de 40 anos, foram analisadas 137.876 internações no período, com 19.492 óbitos (coeficiente médio de mortalidade mensal para a população da idade: 3,7/100.000), com média de permanência hospitalar de 9,2 dias. Não se observou qualquer correlação com a introdução ou suspensão da inspeção, nem variação estatisticamente significativa na série temporal.

Para os acidentes vasculares cerebrais, foram analisadas 69.180 internações, com tempo de permanência médio de 9,2 dias. Houve 10.866 óbitos (média mensal 2,1/100.000 para a população da idade). Não se observou nenhum coeficiente de regressão significativo para a introdução ou a suspensão da inspeção.

Quanto às doenças pulmonares obstrutivas crônicas em pessoas com idade igual ou superior a 60 anos, foram registradas 20.807 internações em 10 anos, com média de permanência de 9,6 dias. Houve 2.627 óbitos, com um coeficiente médio mensal de 1,5/100.000 para a população da idade. Não houve qualquer correlação entre as taxas e a introdução ou suspensão da inspeção veicular.

A evolução temporal dos coeficientes de mortalidade está na Figura 2.

**Figura 2:** Evolução temporal dos coeficientes mensais de mortalidade para a população da idade, por patologias. O período do programa de inspeção está em destaque.

Embora o objetivo principal deste estudo tenha sido a análise de morbidade e mortalidade, foi feito um modelo Arima para os poluentes PM_2,5_ e monóxido de carbono (CO). São mostrados os dados da estação de monitoramento Cerqueira César, na região central do município de São Paulo, a única a ter dados de forma contínua, de todos os anos de 2003 a 2017 no relatório da Companhia Ambiental do Estado de São Paulo (Cetesb)^4^, possibilitando a análise de séries temporais. Para o PM_2,5_ observamos um coeficiente de “tempo” de -0,65 (erro-padrão = 0,22; p = 0,02) no momento da introdução da inspeção, mostrando que a concentração de PM_2,5_ vinha caindo 0,65 μg/m^3^ por ano previamente à inspeção. Nessa estação, não houve nenhuma correlação significativa entre as tendências das concentrações de PM_2,5_ e monóxido de carbono e a introdução ou a suspensão da inspeção (Figura 3).

**Figura 3:** Tendência temporal das concentrações de MP2,5 (em µg/m3) e monóxido de carbono (CO, em ppm, após 2007) conforme monitoração na estação Cerqueira César, região central da cidade de São Paulo. Nota: As linhas de tendência (tracejadas) mostram a tendência de inclinação para baixo prévia ao período da inspeção (sombreado).

## DISCUSSÃO

Este estudo demonstrou, nas séries analisadas, evidências de que o I/M-SP não teve qualquer efeito mensurável sobre a morbidade e a mortalidade hospitalar por doenças respiratórias e do aparelho circulatório no município de São Paulo, nos grupos etários analisados. Em uma estação de monitoração instalada em área central, também foi demonstrado não haver qualquer impacto nos níveis atmosféricos de PM_2,5_ e monóxido de carbono.

A urbanização desordenada da cidade de São Paulo e o aumento de fontes de poluição do ar levaram a uma situação crítica, com concentrações de poluentes regularmente ultrapassando os limites de qualidade mínima^15^. O Programa de Inspeção e Manutenção de Veículos em Uso esteve envolto em controvérsias, desde a contratação sem licitação da empresa responsável pela implantação até o repasse de vultosas verbas públicas, além da falta de transparência nas práticas e resultados. Segundo o último relatório publicado, sobre o ano de 2012, a empresa estimava uma redução de 0,96 μg/m^3^ de PM_2,5_, que teria sido proporcionada pela inspeção dos veículos a diesel, e que com isso teriam sido evitadas 559 mortes por doenças respiratórias^16^. Essas estimativas foram utilizadas como propaganda e tiveram repercussão na imprensa, por terem origem em relatório da Universidade de São Paulo: Saldiva et al.^17^ estimaram que, em 2011, com 75% da frota inspecionada e adequada, a redução de PM_2,5_ emitido por veículos a diesel seria de 28%, resultando em 1,18 μg/m^3^ a menos na concentração para o município. Para estimar o impacto na mortalidade, os pesquisadores utilizaram outra estimativa, da Organização Mundial da Saúde, que considera um impacto de 6% na mortalidade para cada acréscimo de 10 μg/m^3^ na concentração de material particulado fino, chegando-se assim à estimativa de 379 mortes e 894 internações poupadas no município, apenas com a inspeção de 75% da frota diesel^17^. Nenhum desses resultados tem base em medições ou observações, sendo apenas estimativas. No ano de 2011 houve 5.052 óbitos por causas respiratórias no município de São Paulo, em todas as idades^9^. O impacto de 379 óbitos (7,5%) não passaria despercebido, caso existisse.

No estado de São Paulo, são esperadas quase 250 mil mortes relacionadas à poluição até 2030, caso os níveis de PM_2,5_ se mantenham os mesmos que em 2011. Espera-se maior impacto para as doenças do aparelho circulatório e respiratórias em idosos, visto que esse é o grupo que mais cresce na população e é mais vulnerável, assim como os menores de 5 anos^18^. Nas séries temporais que analisamos, não houve variação significativa nas doenças respiratórias em menores de 5 anos no período de 10 anos. Em relação à mortalidade hospitalar por asma em menores de 14 anos, observamos uma tendência de aumento nas taxas no período de 2011 a 2014. O aumento de mortes por asma pode estar relacionado a poluentes, mas mais comumente reflete falta de acesso a medicações e terapias adequadas^19^. Como não houve alteração após a suspensão da inspeção, trata-se mais provavelmente de uma coincidência temporal.

Escolhemos analisar as subpopulações por faixas de idade, devido aos riscos já estabelecidos em inúmeros estudos. Crianças e idosos compõem populações particularmente susceptíveis à exposição a poluentes do ar, com maior evidência de complicações^20^. A escolha das patologias justifica-se pela já bem estabelecida relação entre doenças respiratórias, como a asma e doenças pulmonares obstrutivas crônicas, e o material particulado. A relação fisiopatológica entre exposição ao material particulado e doenças cardiovasculares como o infarto e os acidentes vasculares cerebrais envolve estresse oxidativo, com a presença de marcadores como lipídios oxidados, além da indução de um estado pró-trombótico: a exposição induz a produção de fibrinogênio, fator de von Willebrand e outros fatores de coagulação, além de ativar vias antifibrinolíticas, levando ao aumento da viscosidade plasmática e ativação de plaquetas. A exposição em curto e em longo prazo podem se relacionar também com disfunção endotelial^21^. Existem também fortes correlações epidemiológicas. Gold et al.^22^ demonstraram que o risco de doenças cardiovasculares aumenta de 0,5% a 1,5% para cada 5 a 6 μg/m^3^ de aumento nas concentrações de PM_2,5_. A exposição aguda a PM_2,5_ pode elevar o risco de morte cardiovascular em 69%^22^. A exposição a níveis elevados de monóxido de carbono e PM^2,5^ também pode elevar a pressão arterial^20^_,_ potencializando a ocorrência de acidentes vasculares cerebrais. O risco relativo aumentado de morte por câncer de pulmão, quando relacionado à exposição crônica ao PM_2,5,_ foi observado em metanálise, embora não houvesse relação com a incidência^23^.

Em cidades com predomínio do transporte individual por carro, programas de inspeção e manutenção baseados na “redução das emissões por veículo” tendem a falhar de forma sistemática, pela dificuldade em controlar os três aspectos dessa modalidade de transporte: o veículo, o combustível e o tráfego. Em Guangzhou, na China, cidade com densidade urbana semelhante a São Paulo, duas décadas desses programas não trouxeram nenhuma melhora na qualidade do ar, particularmente em relação a PM_2,5_ e ozônio. De forma semelhante ao ocorrido em São Paulo, políticas públicas estimularam e favoreceram a compra de veículos individuais, e o resultado são concentrações de PM_2,5_ médias de 49 µg/m^3^, quatro vezes superiores às recomendadas^24^. No Brasil, a iniciativa federal Programa de Controle da Poluição do Ar por Veículos Automotores (Proconve) foi o principal responsável pela redução da emissão de poluentes. Iniciado em fases a partir de 1988 e adotando os parâmetros da *US Environmental Protection Agency* (USEPA), o Proconve obteve resultados ao estabelecer para a indústria limites de emissões para veículos de fabricação no país, além de promover o uso em larga escala de biocombustíveis, como o etanol e o biodiesel, e a redução do conteúdo de enxofre dos combustíveis^15^. Em São Paulo, podemos atribuir a essas iniciativas e à renovação da frota o declínio das concentrações de PM_2,5_ e monóxido de carbono, a partir do ano 2000^4^, cujas tendências se observam na Figura 3.

Dentre os motivos pelos quais o I/M-SP não teve impacto sobre os níveis de material particulado na região central e na morbidade e mortalidade, o mais simples se destaca no relatório da empresa responsável: dos 3.037.474 veículos inspecionados em 2012, incluindo motos, carros, ônibus, veículos leves a diesel e caminhões, 97,3% foram aprovados na primeira inspeção, ou seja, de cada 100 veículos, apenas três precisariam de fato ter sido inspecionados pelos critérios adotados, tornando o serviço irrelevante^16^. Não há notícias de que carros reprovados ou não inspecionados tenham sido removidos de circulação, o que poderia ser uma medida com alguma efetividade, embora limitada: mesmo a radical remoção de veículos inadequados e motocicletas teria eficácia transitória, compensada em poucos anos pelo crescimento da frota^25^.

O I/M-SP teve um fim melancólico após custar centenas de milhões de reais à população do município. O Ministério Público de São Paulo ajuizou ação contra os gestores municipais e a empresa prestadora, por “inabilitação técnica, econômica e financeira para executar o contrato, fraudes na mudança do controle acionário e na composição do capital social, inconstitucionalidade de leis municipais sobre a inspeção veicular obrigatória e uma série de outras irregularidades”^26^. Mesmo que em São Paulo a inspeção conseguisse adequar todos os carros, não havendo veículos fora das especificações circulando, ainda restariam a enorme queima de combustíveis fósseis e os congestionamentos. Conforme o relatório da Cetesb, a atual situação da poluição em São Paulo requer também medidas complementares que viabilizem a redução do número de viagens motorizadas e dos congestionamentos, maior oferta de transporte público não poluente e aumento da eficiência do transporte público por ônibus e do transporte de carga, além de melhoria da gestão do sistema viário complementada com ações de planejamento do uso do solo voltadas para a redução do impacto da mobilidade e da logística^4^. É inevitável também argumentar que todos os recursos utilizados na inspeção desnecessária de carros poderiam ser aplicados em medidas de saúde pública, como a distribuição de corticoides inalatórios e acompanhamento regular para asmáticos, ou programas de assistência para os grupos de risco para morbidades específicas.

Uma limitação deste estudo é a sua natureza retrospectiva e observacional. Embora modelos estatísticos possam refletir de forma relativamente acurada a realidade, não é possível neste tipo de estudo nenhum controle sobre as características dos pacientes, e variáveis de confusão não podem ser computadas. Dados armazenados em repositórios públicos, de múltiplas fontes, podem ser heterogêneos em relação à qualidade. No entanto, a enorme quantidade de dados disponível no Datasus permitiu a confecção de um modelo que seguramente reflete o comportamento temporal não estacionário das séries analisadas, tornando o estudo potencialmente útil na avaliação de novas políticas públicas. A avaliação do desempenho de qualquer política de saúde deve estar lastreada na eficiência, transparência e publicidade dos dados.
